# Dedifferentiation, Transdifferentiation, and Proliferation: Mechanisms Underlying Cardiac Muscle Regeneration in Zebrafish

**DOI:** 10.1007/s40139-015-0063-5

**Published:** 2015-01-30

**Authors:** Kazu Kikuchi

**Affiliations:** 1 Developmental and Stem Cell Biology Division, Victor Chang Cardiac Research Institute, Darlinghurst, NSW 2010 Australia; 2 St. Vincent’s Clinical School, University of New South Wales, Kensington, NSW 2052 Australia

**Keywords:** Regeneration, Zebrafish, Cardiomyocyte, Dedifferentiation, Transdifferentiation, Proliferation

## Abstract

The adult mammalian heart is increasingly recognized as a regenerative organ with a measurable capacity to replenish cardiomyocytes throughout its lifetime, illuminating the possibility of stimulating endogenous regenerative capacity to treat heart diseases. Unlike mammals, certain vertebrates possess robust capacity for regenerating a damaged heart, providing a model to understand how regeneration could be augmented in injured human hearts. Facilitated by its rich history in the study of heart development, the teleost zebrafish *Danio rerio* has been established as a robust model to investigate the underlying mechanism of cardiac regeneration. This review discusses the current understanding of the endogenous mechanisms behind cardiac regeneration in zebrafish, with a particular focus on cardiomyocyte dedifferentiation, transdifferentiation, and proliferation.

## Introduction

Myocardial infarction is a leading cause of death worldwide. Urgently needed are the therapies that facilitate replacement of damaged cardiac tissue with new functional cardiomyocytes. In principle, the damaged heart could be regenerated by transplanting cardiomyocytes differentiated in vitro from a variety of cellular sources such as endogenous cardiac stem/progenitor cells [1, 2], embryonic stem cells [3–5], and induced pluripotent stem cells [6]. Alternatively, cardiomyocytes could be directly generated from cardiac fibroblasts in vitro and in vivo by overexpressing defined cardiac transcription factors [7–11] or MicroRNAs (miRNAs) [12].

Another approach would be to identify successful examples of cardiac regeneration in nature, elucidate their underlying mechanisms, and then attempt to apply the insights gained to humans through the provision of the appropriate regenerative stimuli. Urodele amphibians and teleosts are well-known examples of animals that possess remarkable regenerative capacity in a variety of structures and organs as adults [13, 14]. Among these, the zebrafish arguably displays the most robust and best characterized cardiac regenerative responses known till date [15–20]. This review will discuss the mechanism underlying cardiac regeneration in zebrafish with a particular focus on cardiomyocyte dedifferentiation, transdifferentiation, and proliferation.

## Evidence and Mechanisms Underlying Cardiomyocyte Dedifferentiation

**Fig. 1:**
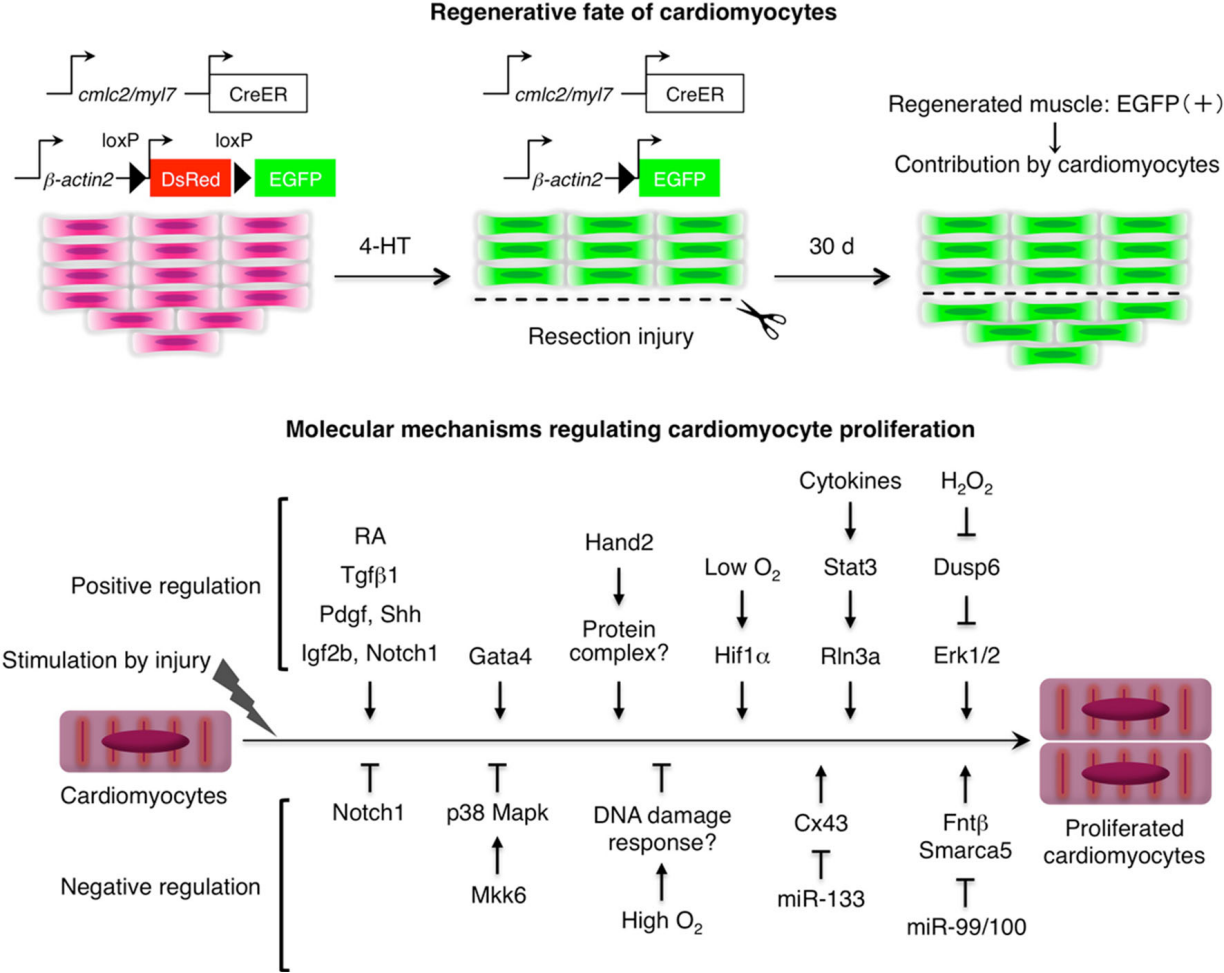
Genetic fate-mapping experiments have provided conclusive evidence that spared cardiomyocytes (EGFP^+^), but not non-myocytes (EGFP^−^), are the major source for new cardiac muscle regenerated at the wound area. After injury, cardiomyocytes are activated and proliferate to regenerate lost myocardium, which is positively and negatively regulated by various intrinsic and extrinsic mechanisms. *4-HT* 4-hydroxytamoxifen

Genetic fate-mapping studies conducted by Poss and Izpisua Belmonte groups have provided convincing evidence that new myocardium is generated from differentiated cardiomyocytes during zebrafish heart regeneration [21••, 22••] (Fig. 1). In both studies, two transgenic zebrafish strains that were essentially the same were used: one line was an inducible Cre line in which the promoter of cardiac myosin light chain 2 (*cmlc2/myl7*) gene drives the myocardial expression of tamoxifen-inducible Cre recombinase (CreER) and the other is an indicator line in which enhanced green fluorescent protein (EGFP) will be expressed in CreER^+^ cells, only when CreER excises a loxP-flanked stop cassette after tamoxifen treatments. In the double transgenic context of these lines, almost all Cmlc2^+^ cardiomyocytes were labeled with EGFP in the uninjured heart by tamoxifen treatments, enabling the examination of the extent of contribution of existing cardiomyocytes to the regenerated cardiac tissue. After the labeling, the hearts were injured at the ventricular apex with resection surgery, removing approximately 20 % of the ventricular muscle, and histologically examined at 30 days post-injury, a timepoint at which regeneration is normally completed. The result clearly showed that the majority of the regenerated myocardium retains EGFP expression, with no significant difference detected in the proportion of the EGFP^+^ muscle in the regenerated tissue compared with that in the uninjured tissue. Thus, existing Cmlc2^+^ cardiomyocytes, but not Cmlc2^−^ non-myocytes, are the major source for new cardiac muscle during zebrafish heart regeneration (Fig. 1). The same result has been observed during the regeneration after genetic ablation of cardiomyocytes [20], in which nearly 60 % of the entire muscle was depleted, as well as during the regeneration of the neonatal mouse heart [23••], suggesting that differentiated cardiomyocytes are the robust source for creating new muscle in natural cardiac regeneration models.

Regenerating cardiomyocytes in the zebrafish heart has unique morphological and molecular signatures distinct from that seen in non-regenerating cardiomyocytes. Histological analyses using transmission electron microscopy and immunofluorescence staining have revealed that cardiomyocytes at the injury border zone acquire less-organized sarcomeres with reduced Z-bands, undergoing DNA synthesis and mitosis [21••, 22••]. Analyses of fluorescence reporter strains have shown that the regulatory sequences of cardiogenic transcription factors such as Gata4 and Hand2 are activated in cardiomyocytes adjacent to the wound area, and such profiles are maintained throughout the regeneration process [21••, 24]. Together with the observation that the expression of other embryonic cardiogenesis genes is also induced in the injured heart [25, 26], these data suggest that zebrafish cardiomyocytes reduce their contractile state upon injury, acquiring immature phenotypes that likely facilitate cell division. Interestingly, the morphological and molecular features described above are reminiscent of those observed in mammalian cardiomyocytes during development. During the development of the mouse heart, disassembly of sarcomere structures have been detected in proliferating cardiomyocytes [27], and the Gata4 gene is expressed in the myocardium and is functionally required for cardiomyocyte proliferation [28], suggesting that mechanisms underlying cardiomyocyte dedifferentiation and proliferation are conserved between zebrafish and mammals.

The notion that molecular programs of zebrafish cardiomyocyte dedifferentiation and proliferation might be conserved in the non-regenerative mammalian heart has been addressed in a recently published study [29]. To identify a conserved mechanism, Aguirre et al. focused on miRNAs, small non-coding RNA molecules that regulate gene expression at post-transcriptional levels, given their wide potential to regulate gene expression changes. They identified that two miRNA families, miR-99/100 and let-7a/c, are sharply downregulated during zebrafish heart regeneration, and they provided evidence that miR-99/100 negatively regulates cardiomyocyte proliferation and regeneration. miR-99/100 targets two proteins: (1) Fntβ, the beta subunit of farnesyl-transferase and (2) Smarca5, a SWI/SNF family chromatin regulator; a pharmacological blockade of Fntβ reduced cardiomyocyte proliferation and impaired regeneration. Unlike the zebrafish heart, the expression of miR-99/100 was unchanged in the mouse heart before and after injury; however, when miR-99/100 was forcefully downregulated in cultured cells, adult mouse cardiomyocytes disassembled sarcomere, re-expressed GATA4, and induced proliferation. These phenotypes were not observed when both miR-99/100 and its protein targets were depleted, indicating that FNTβ and SMARCA5 play a major role in the regulation controlled by the miR-99/100 pathway. Finally, the authors examined the role of the miR-99/100 pathway in the infarcted mouse heart and showed that miR-99/100 and let-7a/c knockdown significantly increases the number of cardiomyocytes with proliferation and dedifferentiation phenotypes, reducing scar size and improving cardiac function. These results indicated that the miR-99/100 pathway is dormant and yet can be reactivated to induce cardiomyocyte dedifferentiation and proliferation in mammalian hearts [29]. It remains unclear how FNTβ and SMARCA5 promote regeneration; however, the study described above supports a concept that deciphering molecular mechanisms underlying cardiac regeneration in zebrafish will provide insights into how endogenous regeneration can be enhanced in mammalian hearts.

## Transdifferentiation and Cardiac Muscle Regeneration

Transdifferentiation is a regenerative phenomenon in which one cell type transforms to another, occasionally via an undifferentiated intermediate, and has been reported as a mechanism observed during natural regeneration in certain vertebrates. A classic example is lens regeneration in adult newts, in which a new lens is formed from the dorsal pigmented iris [30]. Transdifferentiation may also play a critical role in limb regeneration in urodele amphibians. During axolotl limb regeneration, although most cell types maintain their lineages within the blastema, dermis-derived cells re-differentiate into cartilage and tendons likely through transdifferentiation [31]. Little is known about how transdifferentiation is naturally induced upon injury, but a key event during this remarkable process would be the change in the epigenetic state of differentiated cells.

Given our understanding of the epigenetic stability of differentiated states, it seems that such a change would only occur in developmentally related lineages, and therefore, transdifferentiation may not be commonly observed in other regeneration models under natural conditions. However, it is increasingly recognized that epigenetic states can be reprogrammed by manipulating gene regulatory networks, which is most notably shown by the induction of pluripotent stem cells from adult somatic cells by the expression of defined transcription factors [32]. More recent studies have demonstrated that the overexpression of cardiac transcription factors [7–11] or miRNAs [12] can convert non-cardiomyocytes into functional cardiomyocytes.

Transdifferentiation may occur naturally to accommodate extreme loss of a particular cell lineage. When nearly all pancreatic *β* cells are depleted in the adult mouse pancreas, glucagon-producing *α* cells, which would otherwise never change its lineage, are shown to transdifferentiate into insulin-producing *β*-like cells, reverting hyperglycemia to normoglycemia [33]. Chi and colleagues have recently revealed that a similar mechanism contributes to cardiac muscle regeneration in the zebrafish heart [34•]. The authors generated a transgenic line in which ventricular cardiomyocytes can be depleted in a temporally regulated manner and found that zebrafish embryos completely regenerate ventricular myocardium within 4 days after massive ablation of ventricular muscle. To address the mechanism underlying this remarkable regeneration, the authors performed immunofluorescence staining and found that mitotic response is elevated not only in the regenerating ventricle but also in the atrium. Live-imaging and genetic fate-mapping experiments revealed that after the massive ablation of the ventricular muscle, atrial cardiomyocytes migrate into the damaged ventricle, expanding across the chamber, and regenerate ventricular myocardium.

To understand the molecular mechanism underlying the atrial-to-ventricular transdifferentiation, the authors performed gene expression analyses and found that the ventricular muscle depletion activates the re-expression of various heart field marker genes and the ectopic expression of Notch pathway genes in the damaged heart. Pharmacological inhibition of the Notch pathway impaired ventricular muscle regeneration after the ablation. Interestingly, using a reporter zebrafish strain in which cells receiving Notch signaling can be visualized by EGFP expression, the authors observed the activation of Notch signaling in the atrial endocardium, but not in the myocardium during regeneration, indicating that atrial-to-ventricular transdifferentiation is indirectly regulated by Notch signaling through the activation of the atrial endocardium [34•].

The results described above were obtained from zebrafish embryos, and the atrial-to-ventricular transdifferentiation was not observed in the adult heart [34•], which suggests that the transdifferentiation capacity differs depending on age. Interestingly, a recent study using the aforementioned *β* cell ablation model has shown that somatostatin-producing *δ* cells, not the glucagon-producing *α* cells, transdifferentiate into *β*-like cells in 2-week-old mice after massive ablation of *β* cells [35]. Similarly, transdifferentiation to ventricular muscle might occur in the adult heart more prominently from a cell type different from atrial cardiomyocytes. The atrial-to-ventricular transdifferentiation described above depends on the endocardial activation by the Notch pathway [34•]. This mechanism seems reminiscent of the role of the endocardium in the regenerating adult heart, in which endocardial cells produce various paracrine factors, promoting the local proliferation of cardiomyocytes at the regenerating area [26]. Interesting future experiments would comprise the investigation of the factors that are released from the endocardium after Notch activation and identification of a direct mechanism to induce genetic programs for atrial-to-ventricular transdifferentiation.

## Molecular Regulation of Cardiomyocyte Proliferation

Given the identification of existing cardiomyocytes as the dominant source for the regenerated myocardium in the zebrafish heart, the mechanism underlying cardiomyocyte proliferation is increasingly recognized as a major area of interest within the field (Fig. 1). Thus far, a number of paracrine factors have been identified to be released in the regenerating area from the epicardium, the outermost mesothelial tissue, and/or from the endocardium, the innermost endothelial tissue. These factors include retinoic acid (RA) and developmental growth factors such as Tgfβ1, Pdgf, Shh, and Igf2b; the expression of these genes are maintained throughout the regeneration process near the wound area and promote local proliferation of cardiomyocytes [25, 26, 36–39]. The expression of the Notch family genes is also induced at the injury site [40, 41]; interestingly, either inhibiting or activating the Notch pathway similarly blocked cardiomyocyte proliferation and regeneration, suggesting that successful regeneration of the zebrafish heart requires a fine balance between the activation and inhibition of the Notch pathway [41].

As mentioned earlier, the expression of the cardiogenic transcription factor genes is upregulated in regenerating cardiomyocytes [21••, 24–26]. Among these, the function of Gata4 and Hand2 has recently been examined during cardiac regeneration. Gupta et al. overexpressed a dominant-negative form of Gata4 (g4DN) in cardiomyocytes and examined the effect of Gata4 inhibition during cardiac growth and regeneration [42]. When *g4DN* expression was induced in juvenile zebrafish, multiple signs of heart failure, including lethargy, gasping behavior, as well as edema were observed, and the animal survival was sharply reduced to approximately 16 % of the control level; when induced in adult zebrafish, such phenotypes were not observed, suggesting that Gata4 has an essential role for maintaining cardiac function during growth. The authors next performed regeneration experiments after myocardial induction of *g4DN* and found that with *g4DN* expression, myocardial proliferation is reduced and heart regeneration is arrested with collagenous scar formation at the wound area. Interestingly, although *g4DN* was induced in the entire myocardium, the proliferation of cardiomyocytes was normal in the inner trabecular muscle but severely blocked in the lateral ventricular wall muscle [42], where the activation of the *gata4* promoter has been detected [21••], which suggests that the proliferation of trabecular cardiomyocytes is regulated by a Gata4-independent mechanism.

More recently, Yelon and colleagues generated a new transgenic strain that allows tamoxifen-inducible *hand2* expression, in combination with the myocardial specific CreER strain, and examined whether Hand2 overexpression affects the regenerative capacity of cardiomyocytes [24]. When *hand2* expression was induced in the injured heart, no effect was observed in myocardial proliferation; however, when induced after injury, proliferation was significantly elevated in cardiomyocytes in the vicinity of the wound area, indicating that *hand2* overexpression promotes cardiomyocyte production during regeneration. The authors also confirmed that the *hand2* overexpression increases the number of cardiomyocytes in the early zebrafish embryos. Interestingly, this increase was similarly induced by the expression of a Hand2 mutant lacking the DNA-binding domain but not by the one lacking the dimerization domain, suggesting that Hand2 regulates the proliferation of cardiomyocytes by interacting as multimeric complexes [24].

Both Gata4 and Hand2 have been known to have a capacity for converting mammalian cardiac fibroblasts into cardiomyocyte-like cells, when overexpressed with other cardiac transcription factors such as Mef2c and Tbx5 [7–11]. Given that all these factors are upregulated in the regenerating area of the zebrafish heart, it would be interesting to speculate that the injury-induced upregulation of cardiogenic transcription factors might have an instructive role in inducing immature phenotypes in cardiomyocytes during cardiac regeneration.

Regeneration is a phenomenon initiated by injury, and therefore, it appears logical to speculate that key signals to initiate regenerative responses may be provided by immediate events provoked by injury. Given the high oxygen demand in contracting myocardium, tissue hypoxia is a reasonable candidate for an immediate event after injury. Jopling et al. have recently addressed how hypoxia affects heart regeneration in zebrafish [43•]. The authors first performed regeneration experiments under severe anemia, induced by treating fish with a hemolytic chemical reagent, and found that cardiomyocyte proliferation is elevated in anemic zebrafish during regeneration. They next generated a transgenic strain that enables Cre-dependent expression of the dominant-negative form of hypoxia-inducible factor α (dnHIFα), a transcription factor essential for the cellular response to hypoxia, and performed regeneration experiments using the double transgenic fish crossed with a cardiomyocyte-specific CreER strain. With dnHIFα expression, DNA synthesis was reduced in cardiomyocytes and cardiac muscle regeneration appeared to be incomplete at 1 month after the injury, indicating that Hifα-mediated hypoxia signaling is essential for cardiac muscle regeneration in zebrafish. When injured zebrafish were maintained in a hyperoxic condition during the period of regeneration, cardiomyocyte proliferation was decreased and cardiac muscle regeneration was impaired. Together, these results indicated that environmental oxygen concentration regulates cardiomyocyte proliferation in the regenerating zebrafish heart [43•], which is consistent with a recent finding that a higher oxygen concentration in the post-natal environment of the mouse heart limits the proliferation of cardiomyocytes [44•].

Acute inflammatory response is another immediate event induced by injury. Although inflammation appears to be intimately associated with fibrotic responses and regeneration failure observed in most adult mammalian tissues [45], a recent study has demonstrated that acute inflammatory signals can promote regeneration of damaged nervous tissues in adult zebrafish [46]. Yi et al. have recently performed cardiomyocyte-specific unbiased gene expression analyses using regenerating zebrafish heart samples and found that the most prominently upregulated are the genes for inflammatory cytokines and for Jak/Stat3 pathway members, molecules critical for transducing inflammatory signals [47]. The authors generated a transgenic line that enables inducible expression of dominant-negative Stat3 (dnStat3) after the excision of loxP-flanked stop sequences, and crossed this line with the *cmlc2:CreER* line to perform cardiomyocyte-specific loss-of-function analysis of the Stat3 pathway. Regeneration tests using the double transgenic strain revealed that Stat3 inhibition reduced cardiomyocyte proliferation to nearly 20 % of the control level and blocked myocardial regeneration with the formation of a collagenous scar at the wound area. By comparing gene expression profiles obtained from ventricles with or without dnStat3, the authors identified Relaxin 3a (Rln3a), a ligand for a G protein-coupled receptor [48], as a candidate downstream molecule of Stat3 in the regenerating heart. Chromatin immunoprecipitation revealed that Stat3 was recruited to the rln3a promoter region upon cardiac injury, and injection of recombinant human RLN3 partially rescued the impaired proliferation of cardiomyocytes with dnStat3. Thus, during zebrafish heart regeneration, inflammatory cytokines induced by injury likely lead to Stat3 activation in cardiomyocytes, promoting the myocardial release of Rln3a, in turn inducing regenerative proliferation of cardiomyocytes [47].

The reactive oxygen species hydrogen peroxide (H_2_O_2_) has been identified as one of the earliest signals released from damaged tissues to induce inflammatory responses [49]. Xiong and colleagues have recently established a transgenic reporter line with which the H_2_O_2_ level can be monitored and found that H_2_O_2_ production is markedly upregulated in the epicardium near the wound area [50]. Pharmacological inhibition of H_2_O_2_ production as well as transgenic overexpression of catalase, a scavenger enzyme for H_2_O_2_, impaired cardiac muscle regeneration with collagenous scar formation, suggesting that the injury-induced H_2_O_2_ signal is required for regenerative proliferation of cardiomyocytes. Biochemical analyses using cell lines and zebrafish embryos revealed that elevated H_2_O_2_ promotes ubiquitination-dependent protein degradation of Dusp6, a redox-sensitive phosphatase that negatively regulates MAP kinase signaling. Together, these results suggested that H_2_O_2_ plays a critical role in priming cardiomyocyte proliferation after injury by unlocking the suppression of MAP kinase mediated by Dusp6 [50].

Regenerative proliferation needs to be downregulated as the damaged tissue is restored. Although knowledge remains limited, several studies have reported mechanisms underlying this regulation as described below. Consistent with the results obtained from mouse studies, a recent zebrafish study showed that phosphorylated-p38 Mapk is present in the nuclei of non-proliferating cardiomyocytes but disappears when cardiomyocytes enter the mitotic cycle in vitro and in vivo [51]. The myocardial overexpression of the constitutive active form of Mkk6, an upstream kinase of p38 Mapk, was shown to block cardiomyocyte proliferation and arrest cardiogenesis at the wound area.

A mechanism underlying negative regulation of cardiomyocyte proliferation has also been attributed to miRNAs. As described earlier, miR-99/100 represses regenerative proliferation of cardiomyocytes by downregulating the expression of Fntβ and Smarca5 [29]. Unbiased miRNA expression analyses on regenerating hearts have also revealed that the expression of miR-133, a mammalian ortholog of which contributes to cardiac development and diseases [52, 53], inversely correlates with the progress of cardiac regeneration in zebrafish. Constitutive expression of miR-133 was detected in cardiomyocytes in the uninjured heart, and its overexpression was shown to reduce cardiomyocyte proliferation by nearly 50 % and block myocardial regeneration with collagenous scar formation at the wound area. By contrast, the proliferation cardiomyocytes was elevated when miR133 molecules were removed by expressing “sponge” sequences, which contain triplicates of perfect-match binding sites for miR-133. As a novel target of miR-133, the authors identified connexin 43 (Cx43), which is not only widely known as a gap junction protein but has also been reported to regulate cell proliferation and growth, and showed that the pharmacological inhibition of cx43 function blocks regenerative proliferation of cardiomyocytes. Together, these results indicated that miR-133 negatively regulates cardiomyocyte proliferation, partly by modulating the Cx43 level, during cardiac regeneration in zebrafish [54].

## Conclusion

Zebrafish hearts regenerate mainly through cardiomyocyte dedifferentiation and proliferation [21••, 22••], a mechanism also underlying the regeneration of the neonatal mouse heart [23••]. The regenerative capacity of the neonatal mouse heart diminishes within a week, seemingly concomitant with cardiomyocyte binucleation [55]; however, even after this period, robust proliferation has recently been shown to occur in binucleated cardiomyocytes at post-natal day 15, establishing the final number of cardiomyocytes in the adult heart [56••]. Cardiomyocytes in the adult mammalian heart do not appear to have a significant capacity for proliferation in the injury setting, but they maintain a measurable capacity for self-renewal throughout life [57••, 58••]. A recent result supports the notion that mechanisms underlying regeneration of the zebrafish heart are conserved in mammalian hearts and can be reactivated to induce regeneration [29]. Further understanding of the cellular and molecular regulation of cardiac regeneration in zebrafish will provide valuable insights for a better understanding of how the human heart can be stimulated to reactivate its endogenous regenerative capacity as a novel therapeutic strategy to treat cardiac diseases.
